# Use of the Stockwell Transform in the Detection of P300 Evoked Potentials with Low-Cost Brain Sensors

**DOI:** 10.3390/s18051483

**Published:** 2018-05-09

**Authors:** Alan F. Pérez-Vidal, Carlos D. Garcia-Beltran, Albino Martínez-Sibaja, Rubén Posada-Gómez

**Affiliations:** 1 Tecnológico Nacional de México-CENIDET, Interior Internado Palmira S/N, Col. Palmira, Cuernavaca, Morelos, C.P. 62490, México; cgarcia@cenidet.edu.mx; 2 Tecnológico Nacional de México-Instituto Tecnológico de Orizaba, Av. Oriente 9 N° 852, Col. Emiliano Zapata, Orizaba, C.P. 94320, México; amartinez@ito-depi.edu.mx (A.M.-S.); rposada@itorizaba.edu.mx (R.P.-G.)

**Keywords:** P300 evoked potentials, Stockwell transform, electroencephalograph, brain-computer interface, non-invasive brain sensors, signals processing, wireless device

## Abstract

The evoked potential is a neuronal activity that originates when a stimulus is presented. To achieve its detection, various techniques of brain signal processing can be used. One of the most studied evoked potentials is the P300 brain wave, which usually appears between 300 and 500 ms after the stimulus. Currently, the detection of P300 evoked potentials is of great importance due to its unique properties that allow the development of applications such as spellers, lie detectors, and diagnosis of psychiatric disorders. The present study was developed to demonstrate the usefulness of the Stockwell transform in the process of identifying P300 evoked potentials using a low-cost electroencephalography (EEG) device with only two brain sensors. The acquisition of signals was carried out using the Emotiv EPOC^®^ device—a wireless EEG headset. In the feature extraction, the Stockwell transform was used to obtain time-frequency information. The algorithms of linear discriminant analysis and a support vector machine were used in the classification process. The experiments were carried out with 10 participants; men with an average age of 25.3 years in good health. In general, a good performance (75–92%) was obtained in identifying P300 evoked potentials.

## 1. Introduction

In recent times, technological progress has allowed brain-computer interfaces (BCI) to be used more frequently. Their main purpose is to control devices by means of brain signals. This is of great relevance in the area of rehabilitation because it provides a different method of communication for those who have a motor disability, such as amyotrophic lateral sclerosis, Becker muscular dystrophy, Duchenne muscular dystrophy, Guillain-Barré syndrome, quadriplegia, brain injury, spinal cord injury, and so forth. BCI systems have been developed that allow the detection of mental fatigue [1,2], movement of joints [3], imaginary movement [4,5,6], mental tasks [7], emotions [8], and more. EEG signals are electrical potentials caused by a set of neurons when a brain process is performed. They are obtained using an electroencephalograph, directly from the scalp. These signals are considered stochastic because they have great variability and a low signal-to-noise ratio. At present, several types of EEG signals have been classified, such as the sensorimotor rhythm (SMR) [9], slow cortical potential (SCP) [10], event-related potential (ERP) [11], and steady-state visual evoked potential (SSVEP) [12], among others.

The P300 wave is an ERP which is associated with cognition. It is a positive deflection of the electric potential which is generated approximately 300–500 ms after an infrequent stimulus related to a specific event [13]. It is most evident in the delta and theta frequency bands [14,15]. The stimuli can be visual [16], auditory [17], or somatosensory [18]. It has been shown that the less probable the stimulus, the greater the amplitude of the response peak [19]. The P300 evoked potential has been used in applications such as lie detectors, spellers, and the diagnosis of psychiatric disorders [20]. 

The P300 speller is one of the most commonly-used applications in BCI systems. This application allows the selection and display of different characters on a digital screen through the detection of P300 evoked potentials generated from visual stimuli. It was proposed by Farwell and Douchin in 1988 [21]. It has been reported that the electrodes P07, P08, Fz, Cz, Pz, and Oz are efficient in detecting P300 evoked potentials, with these regions being associated with memory, attention, and visual processes [22]. 

Currently there are several methodologies that allow the detection of P300 evoked potentials [23,24,25]. Algorithms such as the wavelet transform have extracted patterns of the EEG signal in a time-frequency distribution, performing excellently in classification [13]. However, most of the methodologies developed use high-resolution professional EEG equipment with several acquisition electrodes, which is not feasible for some institutions that do not have the necessary resources to acquire this equipment. Recently, technological advancement has allowed the development of portable EEG devices that are economical compared to professional EEG equipment. By using these portable devices, lower quality EEG signals with greater noise are acquired. For this reason, it is necessary to use appropriate methods to obtain an optimal classification performance.

In this study, different algorithms were investigated to define those that are more efficient at classifying P300 evoked potentials obtained using Emotiv EPOC^®^, a wireless EEG device. This equipment was manufactured by the company EMOTIV located in San Francisco, USA. The Stockwell transform was used as a feature extractor, as other studies have reported it has a good time-frequency resolution and is extensively used for the analysis of non-stationary signals, such as EEG signals [26]. In addition, the classifiers of linear discriminant analysis (LDA) and the support vector machine (SVM) were used, of which the SVM classifier performed better in the detection of P300 evoked potentials.

This article is organized as follows: Section 2 describes some works related to BCI systems, Section 3 details the characteristics of the Stockwell transform, Section 4 outlines the materials and methods used in this study, Section 5 analyzes the data and results and, finally, in Section 6 the discussion is presented.

## 2. Related Work

Some important features of the EEG signal are hidden in the time domain, so certain investigations are needed to analyze the signal in the frequency domain [27,28]. The Fourier transform is a useful tool in the study of stationary signals. It converts a signal that is in the time domain into its frequency equivalent. The Fourier transform is defined as:

(1) $$X(f)=\int_{-\infty }^{+\infty }{h(t)e}^{-j2\pi \mathrm{ft}}\;\mathrm{dt},$$

where h(t) represents the signal in the time domain, f the frequency, t is the time, and X(f) is the signal in the frequency domain. In the paper by Güneysu et al. [29] SSVEP were induced by groups of light emitting diodes operating at different frequencies (7, 9, 11, and 15 Hz). The subjects were focused on a specific group, then with the fast Fourier transform (FFT) and a Gaussian model the dominant frequency component was detected. The performance in terms of detection was 75% on average. In another study [30] the FFT was used for the detection of mental commands, in order to control a wheelchair, and the performance obtained was 76% on average.

The drawback of the Fourier transform is that it does not contain temporal information. Therefore, its use is not recommended for the analysis of non-stationary signals, where the frequency varies with time. The short-time Fourier transform (STFT) solves this problem by adding a window function to the Fourier transform. This provides a local spectrum that allows the analysis of the frequency in different time intervals. The equation is defined as follows:

(2) $$\mathrm{STFT}(\tau ,\;f)\;=\int_{-\infty \;}^{+\infty }h\left(t\right){g(\tau \;-\;t)e}^{-\;j2\pi \mathrm{ft}}\;\mathrm{dt},$$

where h(t) is the signal, f is the frequency, and g(τ − t) is the window function. In the paper by Phothisonothai et al. [31], through the STFT it was found that the coherence difference in the theta and alpha bands was statistically significant, and the duty cycle was suggested as a characteristic for SSVEP-based applications. The main drawback of the STFT is that the resolution in time and frequency remains constant. This is because the extension of the window function remains fixed throughout the analysis.

The wavelet transform is used in the analysis of non-stationary signals. It represents the signal in its frequency components during a time interval. It is formed by a base function (mother wavelet) that can be modified in its scale and translation factor. The continuous wavelet transform (CWT) is defined as follows:

(3) $$W\left(s,\;\tau \right)=\int_{-\infty }^{+\infty }h(t)\frac{1}{\sqrt{s}}\psi \left(\;\frac{t\;-\;\tau }{s}\right)\;\mathrm{dt},$$

where h(t) represents the signal, ψ(t) is the mother wavelet function, s is the scale factor, and τ is the translation factor. The function ψ(t) expands when s > 1 and contracts when s < 1. The relationship between scale and frequency is inverse; high scales correspond to low frequencies and low scales correspond to high frequencies.

The wavelet transform has been used in several studies for the detection of P300 evoked potentials. In the paper by Motlagh et al. [32] the P300 components were detected with an efficiency greater than 90%. The continuous and discrete wavelet transform were used as feature extractors and the SVM algorithm as a classifier. The EEG signals used were extracted from the database “BCI competition 2003”.

In research by Guo et al. [13] the discrete wavelet transform was used together with the Fisher criterion, obtaining a yield higher than 90% in the detection of P300 evoked potentials. In the paper Costagliola et al. [33] different mother wavelets were compared and it was concluded that the functions daubechies 4, biorthogonal 2.4, biorthogonal 4.4, biorthogonal 5.5, coiflets 2, symlets 4, and symlets 6 provide greater efficiency in the detection of P300 components.

The Gabor transform is used to estimate the time-frequency distribution of a signal. It has been used in the analysis of EEG signals with patients with epilepsy [34]. The equation is defined as follows:

(4) $$G\left(\tau ,\;f\right)=\int_{-\infty }^{+\infty }h\left(t\right)w(\tau \;-{\;t)e}^{-\;j2\pi \mathrm{ft}}\;\mathrm{dt},$$

where h(t) is the signal and f is the frequency. The expression w(τ − t) represents a window function that modifies its extension according to the values of the variance σ:

(5) $$w\left(t\right)=\frac{1}{\sqrt{{2\pi \sigma }^{2}}}{\;e}^{\frac{-t^{2}}{{2\sigma }^{2}}}$$

The Stockwell transform has been used in image processing and in the biomedical field [35]. In research by Senapati et al. [36] and Upadhyay et al. [37] it was shown to be an efficient tool for removing ocular artifacts from EEG signals. In the paper by Vijean et al. [7] it was found to be effective in detecting mental tasks.

## 3. Stockwell Transform

The Stockwell transform allows the analysis of a signal in a time-frequency distribution. It has been shown to have a better resolution than the Gabor transform [38]. It is defined as:

(6) $$S\left(\tau ,\;f\right)=\int_{-\infty }^{+\infty }h\left(t\right)\;w(\tau \;-{\;t,\;f)\;e}^{-\;j2\pi ft}\mathrm{dt},$$

where h(t) represents the signal and w(τ − t, f) is generally defined as a normalized and positive Gaussian function [36]:

(7) $$w\left(\tau \;-\;t,\;f\right)=\frac{|f|}{\sqrt{2\pi }}e^{-\;\frac{{\left(\tau \;-\;t\right)}^{2}f^{2}}{2}}$$

where the window function w(τ − t, f) is shortened as the frequency increases and lengthens when the frequency decreases. The Stockwell transform provides a frequency-dependent resolution and maintains a direct relationship with the Fourier spectrum. Therefore, it obtains local phase information with absolute reference [39].

The phase “with absolute reference” means that the phase information is always referenced to time t = 0. This condition occurs in each of the samples obtained from the Stockwell transform. The average time of the Stockwell transform is equal to the Fourier spectrum [40]. 

One of the drawbacks of the Stockwell transform is the redundant information of the time-frequency space that it generates, which causes greater consumption of computational resources.

## 4. Materials and Methods

### 4.1. Data Acquisition

The EEG signals of this study were acquired in the electronics department of the Centro Nacional de Investigación y Desarrollo Tecnológico (CENIDET). The average age of the 10 participants was 25.3, and all were men in a good state of health. The same experimental conditions were established for each subject so that the same number of samples was obtained from each of them.

**Figure 1:**
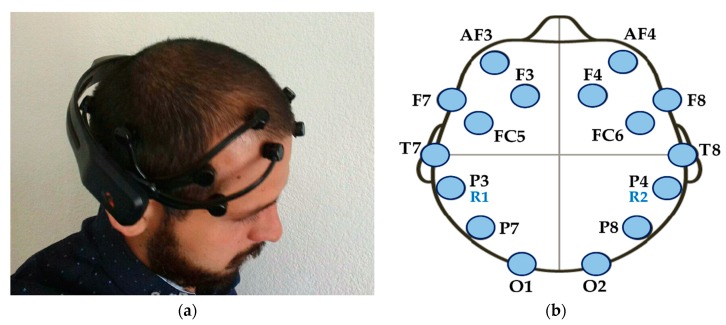
Emotiv EPOC^®^ wireless EEG headset: (**a**) Placement of the device on the head of the subject; (**b**) Distribution of the electrodes according to the international 10–20 system.

The Emotiv EPOC^®^ commercial electroencephalograph was used, which contains 16 electrodes positioned according to the international system 10–20. The EEG signals are obtained from 14 sensors located in the areas AF3, F7, F3, FC5, T7, P7, O1, O2, P8, T8, FC6, F4, F8, and AF4. The other two sensors are used as references and are located in zones P3 and P4. Figure 1 shows the device and the distribution of its electrodes.

The EEG signals obtained had a sampling frequency of 128 Hz and only the electrodes O1, O2 and the references (R1 and R2) were used. The OpenViBE^®^ [41] software version 0.17.1 was used to obtain the P300 evoked potentials. This software allows the manipulation of experimental scenarios such as the P300 speller. In this study, we used the application known as “P300: Magic Card^®^” [42], which is similar to the P300 speller, except that images are displayed instead of characters. 

**Figure 2:**
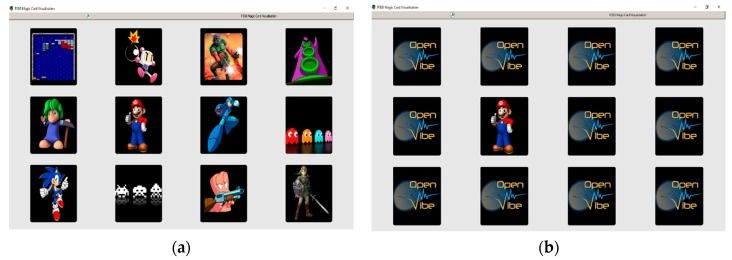
Matrix of images used to obtain P300 evoked potentials: (**a**) At the beginning of the experiment, all the images are displayed; and (**b**) during the development of the experiment, the images are hidden and appear randomly one by one.

During the development of the experiment, each participant was asked to sit in front of the monitor at a distance of 1 m. Then, an array of images in three rows and four columns was projected onto the screen. The matrix of images is shown in Figure 2.

**Figure 3:**
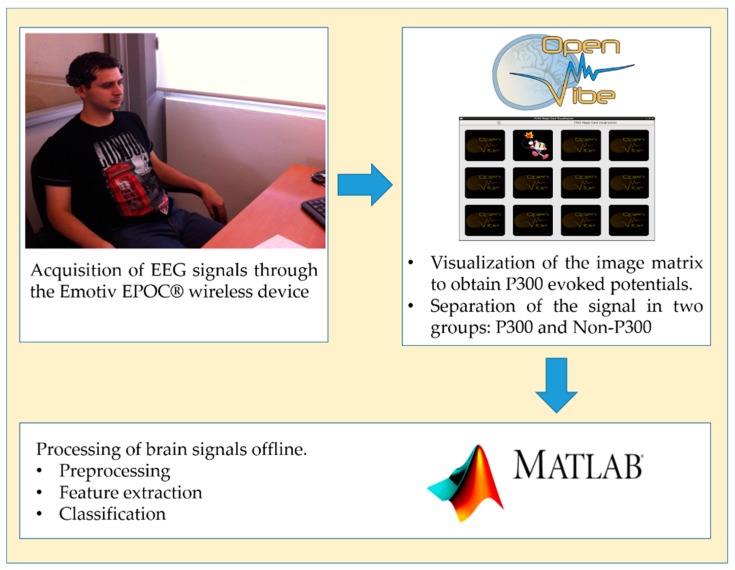
Complete process of the BCI System.

At the beginning of each trial, the subject was asked to focus his attention on a particular image. Then, these images began to appear and disappear at random. In one trial each image appeared 12 times with a fixed time of 200 ms. There was a recess period of 100 ms between each image. The complete experiment consisted of six trials, in total, and each image of the matrix appeared 72 times. This means that each subject generated a total of 72 evoked potentials. Each participant was asked not to blink or perform eye movements as much as possible to avoid generating noise in the EEG signal. In a previous work [43], this same method of P300 evoked potential acquisition was used and obtained excellent results. The complete EEG signal was acquired during an online process through the OpenViBE^®^ software. Then, in an offline process, the OpenViBE^®^ software was used again to divide the signal into two groups. In Group 1 the signals were related to P300 type events, and in Group 2 the signals were not related to this type of event. To obtain Group 1, samples of the EEG signal were taken (epochs of 700 ms) at each instant that the target image was presented. The rest of the signal was considered Group 2. Then, the data was stored for offline processing with Matlab^®^ software version R2012a. Figure 3 shows the complete process of the BCI system development.

### 4.2. Feature Extraction

From the acquired EEG signal, the mean was subtracted. Then, the Stockwell transform was used to obtain a time-frequency distribution of the EEG signal. Subsequently, the samples obtained were divided into different frequency bands: 1–5 Hz (delta), 5–8 Hz (theta), 8–15 Hz (alpha), 15–30 Hz (beta), and 30–64 Hz (gamma). This is in order to compare the success rate of identification in each of these intervals. Each of the frequency bands was averaged and five signals representative of the different cerebral rhythms (delta, theta, alpha, beta, and gamma) were obtained. Then, the signals were divided into time intervals (2 s duration and 0.25 s displacement). Subsequently, different mathematical functions were applied, obtaining different feature vectors.

The mathematical functions used were the standard deviation, kurtosis, asymmetry coefficient, area under the curve, and average power. The different feature vectors obtained were used in the training and classification phase.

### 4.3. Classification

In this study, the classifiers of the linear discriminant analysis (LDA) and the support vector machine (SVM) were used. LDA is one of the most commonly used classification algorithms in BCI systems [44,45], as it is a simple but accurate method for the identification of EEG signals. The LDA algorithm determines the optimal axes in terms of classification by increasing the variance between the classes and decreasing the variance within the class [46]. 

The SVM algorithm is robust in binary classification and is considered one of the most accurate classifiers to detect P300 evoked potentials [25]. The SVM separates the data from two classes by finding a hyperplane with the maximum possible margin [47]. SVM can use different kernel functions, the most used are [48]:

- Radial Basis Function, ${K(x}_{i\;}{,\;x}_{j}{)\;=\;e}^{-\;\frac{\left\|x_{i\;}{\;-\;x}_{j}{}^{2}\right\|}{{2\sigma }^{2}}},\;\sigma \;\neq \;0.$
- Polynomial, ${K(x}_{i\;}{,\;x}_{j}{)\;=\;(x}_{i}{\;\times \;x}_{j}{\;+\;1)}^{d},\;d\;>0.$
- Sigmoidal, ${K(x}_{i\;}{,\;x}_{j}{)\;=\;\tanh(kx}_{i}{\;\times \;x}_{j}\;-\;\delta ).$
- Cauchy, ${K(x}_{i\;}{,\;x}_{j})\;=\;{\left(1\;+\;\frac{{\left\|x\;-\;y\right\|}^{2}}{{2\sigma }^{2}}\right)}^{-1},\;\sigma \;\neq \;0.\;$
- Logarithmic, ${K(x}_{i\;}{,\;x}_{j}{)\;=\;-\;\log(\|x\;-\;y\|}^{d}\;+\;c),\;d\;>0.$

In the classification process only 100 s of the signal acquired in the experiment was used (50 s from each group). The feature vector that was obtained was divided into two equal parts: training and testing. Then, the training vector was divided into two groups (P300 and non-P300), which was used to train the classifier. 

**Figure 4:**
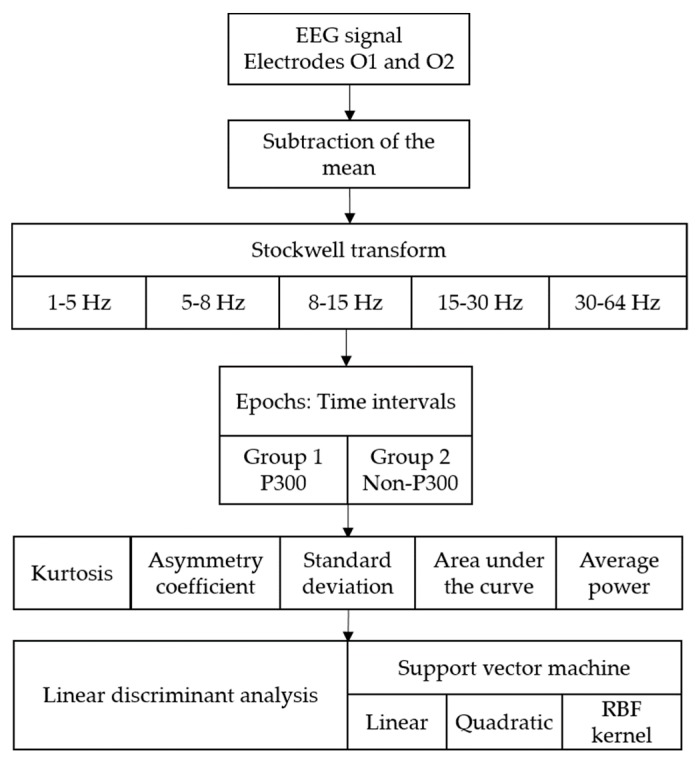
Methodology used in the processing of EEG signals.

The test vector was formed with samples of type P300 and non-P300 distributed alternately (10 segments of 5 s). It was used to verify the efficiency of the classifier. The performance was established according to the number of samples correctly classified in the P300 and non-P300 groups with the LDA and SVM classifiers. The kernel functions used in the SVM were linear, quadratic, and radial basis. In the Gaussian radial base kernel function (RBF), a scale factor (sigma) of 1 and a penalty parameter (C) of 1 were used. The complete methodology with the different algorithms used in this study is shown in Figure 4.

## 5. Results

**Figure 5:**
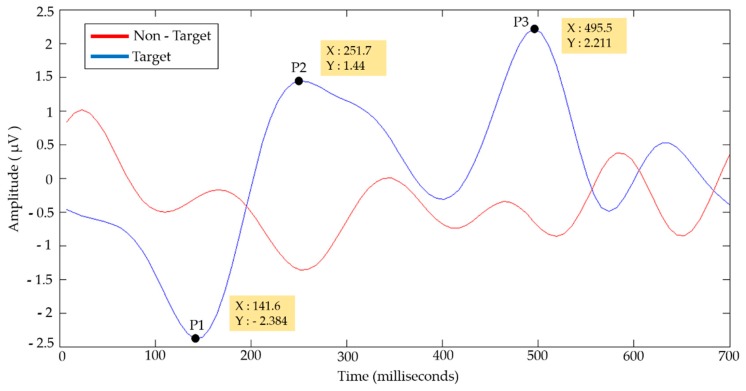
Average EEG signals for the two conditions (Target/Non-Target).

The results showed a higher classification performance in the frequency ranges of 1–5 and 5–8 Hz, corresponding to the delta and theta rhythms. Figure 5 shows the averaged EEG signals of the Subjects for the two conditions (Target/Non-Target).

The average Target signal shows a negative component (P1) with a minimum value of −2.384 μV at 141.6 ms and two positive components (P2 and P3) with maximum values of 1.44 and 2.211 μV at 251.7 and 495.5 ms, respectively. The EEG signals shown were filtered into a frequency band of 1-8 Hz. Table 1 shows the average of the peak values of the components P1, P2 and P3 obtained at different times of occurrence. The Target signals (epochs of 700 ms) that did not clearly present these components were discarded in the average.

**Figure 6:**
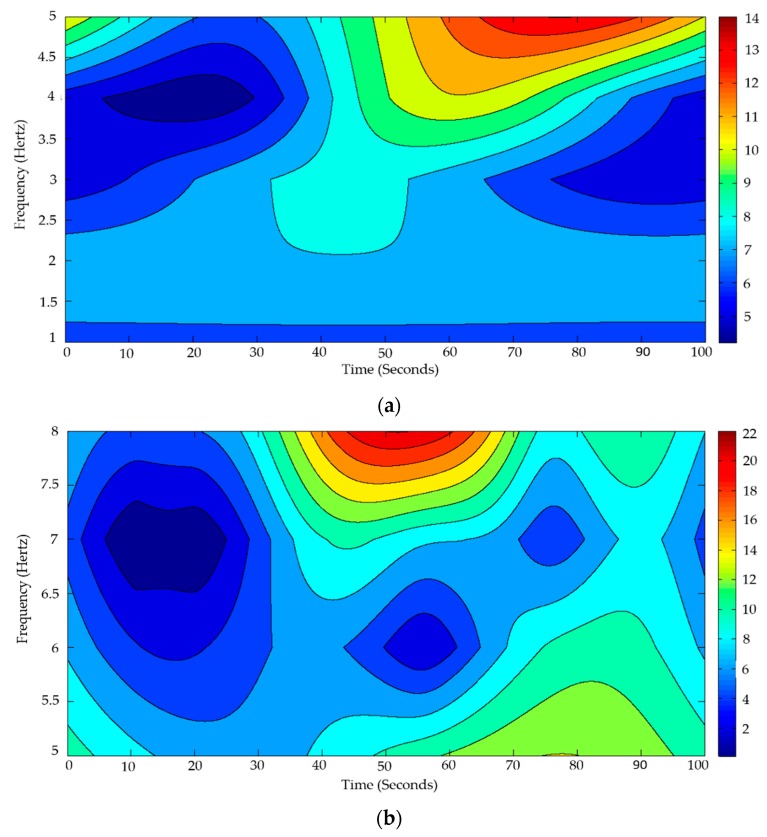
Stockwell transform spectrograms of the EEG signal of Subject 3: (**a**) frequency range of 1–5 Hz; and (**b**) frequency range of 5–8 Hz.

The average amplitudes obtained were lower for P1 and higher for P2 and P3 compared to the average amplitudes of Figure 5. The standard deviations of the amplitudes (4.03, 3.26, and 4.17 μV) show that consistent values were obtained in the peaks of the components P1, P2, and P3, respectively. By means of the standard deviations of the times of occurrence of the amplitude peaks, the time ranges of 113.28–205.24, 203.14–329.2, and 402.12–555.18 ms were established for the components P1, P2, and P3, respectively. Component P3 shows a positive deflection of the electric potential in a time range of 402.12–555.18 ms, which are properties of the P300 evoked potential. Figure 6 shows the Stockwell spectrograms in the frequency ranges of 1–5 and 5–8 Hz obtained from the EEG signal of Subject 3.

The Y-axis represents the frequency distribution (Hertz) and the X-axis represents the time in seconds. Figure 6a shows a frequency range of 1–5 Hz representing the delta rhythm and Figure 6b shows a frequency range of 5–8 Hz representing the theta rhythm. Stockwell transform spectrograms were obtained from an EEG signal distributed alternately in 10 second segments with samples of P300 and non-P300. The color bar represents the instantaneous amplitude obtained by calculating the absolute value of the Stockwell transform. The blue color represents the low amplitudes and the red color the high amplitudes.

**Figure 7:**
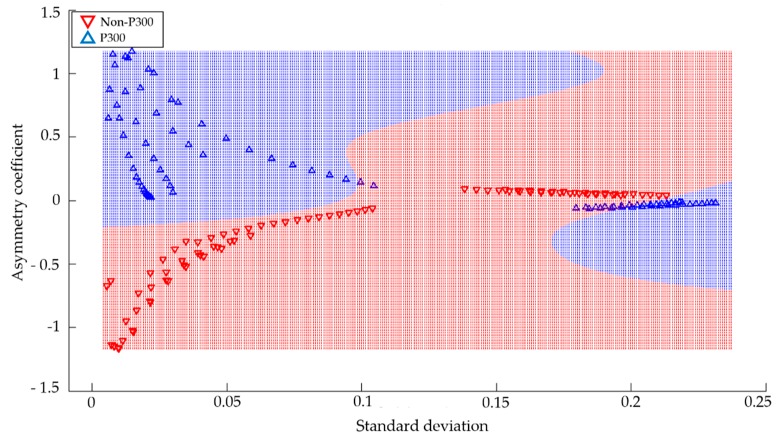
Classification obtained from Subject 3 with the SVM algorithm using the RBF kernel.

The feature vectors that obtained a better performance in the classifiers were the combinations of average power and area under the curve, and the asymmetry coefficient and standard deviation. Finally, the classifier with the best performance was SVM with the RBF kernel. The results shown in this study are based on the parameters that achieved the best performance. Figure 7 shows the result of the classification obtained from Subject 3, in the frequency range of 5–8 Hz, using the functions of asymmetry coefficient and standard deviation with the SVM algorithm.

**Figure 8:**
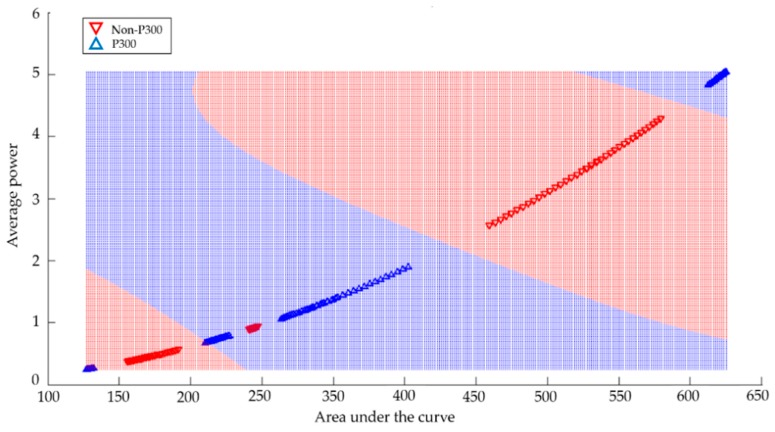
Classification obtained from Subject 2 with the SVM algorithm using the RBF kernel.

In this particular case, 92% accuracy of identification was obtained. The SVM algorithm divided the space into two groups by means of the training feature vectors. The blue area represents the P300 group and the red area represents the non-P300 group. Moreover, triangles of blue and red are displayed, which represent the test feature vectors of the groups P300 and non-P300, respectively. Figure 8 shows the results of the classification obtained from Subject 2 in the frequency range of 5–8 Hz, using the functions of average power and area under the curve with the SVM algorithm.

In this particular case, 80% accuracy was obtained in identification. The data obtained could not be separated efficiently with a linear kernel. Therefore, to correctly separate the two classes of P300 and non-P300 (blue and red color, respectively) the RBF kernel was used with the data to create non-linear combinations of the original features to project them onto a higher dimensional space through a mapping function where it becomes linearly separable. The RBF kernel was used in each of the cases because it allowed a better separation of the two groups. Table 2 shows the methodologies that obtained the best performances with the different subjects.

The classification yields obtained for each of the subjects show that the parameters and algorithms used can correctly identify the P300 evoked potentials. The performance obtained in the frequency range of 1–5 Hz with the feature vectors of average power and area under the curve was 79.5% on average, and with the feature vectors of the asymmetry coefficient and standard deviation was 83.1% on average. 

On the other hand, a better performance was obtained in the frequency range of 5–8 Hz with the feature vectors of the asymmetry coefficient and standard deviation (84.1% on average) than with the feature vectors of average power and area under the curve (80.5% on average). The highest percentages of classification were obtained in the frequency of 5–8 Hz, with the feature vectors of the asymmetry coefficient and standard deviation in Subjects 2 and 3 obtaining values of 90% and 92%, respectively. 

## 6. Discussion

This study shows that the Stockwell transform is a useful algorithm that allows the detection of P300 evoked potentials induced by visual stimuli. Identification was achieved with a commercial wireless electroencephalograph using only the channels O1 and O2. The electrodes were chosen based on other studies [22,49] which showed that the channels Fz, Cz, Pz, PO7, PO8, and Oz contain information that provides a better classification performance in the P300 speller. The Emotiv Epoc^®^ device does not have any of the aforementioned electrodes; however, it has the channels O1 and O2, which are very close to the Oz channel of the 10–20 system. It is also important to mention that the chosen channels are located in the occipital area of the brain, which is associated with visual processes. 

In general, an acceptable classification performance was obtained with the different subjects and the selected methods (above 75%). The highest percentage of classification obtained was 92%. It should be mentioned that other studies that involve the detection of P300 evoked potentials have obtained similar or greater performances; however, most of them use high-cost professional EEG equipment and several acquisition electrodes [50,51,52]. This is an important limitation in the development of research and applications of BCI systems, because institutions do not always have sufficient resources to obtain this equipment. For this reason, the Emotiv EPOC^®^ device was used, which is a low-cost portable device. However, the signals obtained were of poorer quality and, therefore, had a lower signal-to-noise ratio. In addition, it is important to note that this portable device has a limited number of electrodes in a fixed distribution. Due to these deficiencies and limitations, it was necessary to implement a methodology that largely excluded noise, correctly extracted signal characteristics, and achieved an efficient identification of P300 evoked potentials. 

Therefore, a time-frequency analysis was chosen, because these are widely used in the analysis of non-stationary signals. A good time-frequency distribution will only be possible if a narrow window function is used during the analysis of high-frequency components in the signal, and a wider window function is used during the analysis of low-frequency components in the signal. The window function of the Stockwell transform fulfills the previously-mentioned requirements of a good time-frequency distribution [38]. The Stockwell transform is a method of spectral localization that can be considered a generalization of STFT and an extension of CWT [20]. It also has a better resolution than the Gabor transform [38]. Due to this, the development of the proposed BCI system included as a feature extractor the Stockwell transform, and it was shown that this technique allows the correct identification of P300 evoked potentials, even with low-cost equipment and when only acquiring EEG signals from electrodes O1 and O2.

The frequency ranges that allowed better identification of the P300 evoked potentials were within the values of 1–5 and 5–8 Hz. This suggests that the P300 evoked potentials occur in the delta and theta brain rhythms. This has been found in other studies, and in papers by Kolev et al. [53] and Yordanova et al. [54] it was demonstrated by means of a time-frequency analysis that sub-components in the delta and theta bands coexist in the formation of P300 potentials.

In this study, the SVM classifier performed better than the LDA classifier, which suggests that the SVM algorithm is more suitable for the detection of P300 evoked potentials. SVM is an algorithm that allows pattern recognition and provides an excellent solution for discrimination between two different classes. In Thulasidas’s work [55] it was used as a classifier in the P300 speller and high levels of yield were obtained. In the paper by Tayeb et al. [25] it is mentioned that several classification algorithms have been used for the detection of P300 evoked potentials, such as artificial neural networks, naive Gaussian Bayes, and the SVM. Among them, the SVM algorithm is one of the most precise.

In conclusion, an adequate performance (in the range of 75–92%) was obtained in the detection of P300 evoked potentials by means of the Stockwell transform and using low-cost wireless EEG equipment with only two acquisition channels. For future work, a multidimensional analysis will be done with EEG signals from different electrodes. Algorithms, such as the two-dimensional Stockwell transform, will be used to improve performance in the identification of P300 evoked potentials.

## Figures and Tables

**Table 1:** Statistical values of the components P1, P2, and P3.

Component	Amplitude (µV)		Time (ms)	
	Mean	Standard Deviation	Mean	Standard Deviation
P1	−6.29	4.03	159.26	45.98
P2	5.62	3.26	266.17	63.03
P3	8.72	4.17	478.65	76.53

**Table 2:** Performance obtained in the classification process with the SVM algorithm (%).

Subject	Average Power—Area under the Curve		Asymmetry Coefficient—Standard Deviation	
	1–5 Hz	5–8 Hz	1–5 Hz	5–8 Hz
S1	85	81	84	85
S2	81	80	82	90
S3	84	84	80	92
S4	75	76	87	78
S5	77	80	83	82
S6	78	81	86	81
S7	80	75	82	80
S8	79	83	84	86
S9	75	82	81	82
S10	81	83	82	85
Average	79.5	80.5	83.1	84.1
